# Dipentyl 2,6-di­amino­benzo[1,2-*b*:4,5-*b*′]di­furan-3,7-di­carboxyl­ate

**DOI:** 10.1107/S160053681302480X

**Published:** 2013-09-12

**Authors:** Giuseppina Roviello, Fabio Borbone, Antonio Carella, Giovanni N. Roviello, Angela Tuzi

**Affiliations:** a Dipartimento di Ingegneria, Università di Napoli ’Parthenope’, Centro Direzionale di Napoli, Isola C4, 80143 Napoli, Italy; b Dipartimento di Scienze Chimiche, Università degli Studi di Napoli ’Federico II’, Complesso di Monte S. Angelo, Via Cinthia, 80126 Napoli, Italy; c Istituto di Biostrutture e Bioimmagini, CNR, Via Mezzocannone 16, 80134 Naples, Italy

## Abstract

The title compound, C_22_H_28_N_2_O_6_, crystallizes with one half-mol­ecule in the independent unit, the mol­ecule being located on an inversion centre. The penthyl groups are in the all-*trans* conformation and an almost planar conformation of the whole mol­ecule is observed [maximum deviation from the least-squares plane through all non-H atoms is 0.0229 (17) Å for an N atom]. The amino groups are involved in intra- and inter­molecular hydrogen bonds. Intra­molecular hydrogen bonding involving the amino group and ester carbonyl helps to lock the *syn* conformation of the ester with respect to the amino group. In the crystal, N—H⋯O hydrogen bonding involving the amino group and the furan and ester carbonyl O atoms self-assembles the mol­ecules into a two-dimensional hydrogen-bonded network parallel to (010) that displays inter­digital packing sustained by alk­yl–alkyl inter­actions.

## Related literature

For the synthesis and properties of amino­benzodi­furane derivatives, see: Caruso *et al.* (2009 ▶). For O- and N-rich aromatic heterocycles, see: Roviello *et al.* (2007 ▶, 2012 ▶). For mol­ecules with optical and opto-electronical properties, see: Carella *et al.* (2012 ▶); Centore *et al.* (2007 ▶); Roviello *et al.* (2009 ▶); Ricciotti *et al.* (2013 ▶); Vitaliano *et al.* (2009 ▶). For hydrogen bonding in heterocycles, see: Centore *et al.* (2013*a* ▶,*b* ▶).
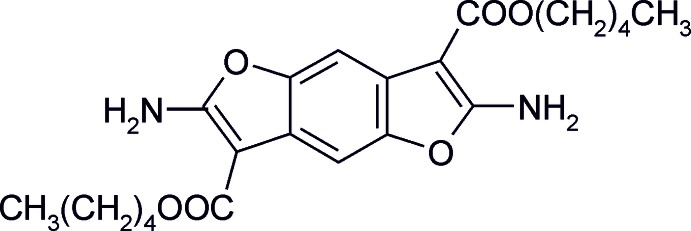


## Experimental

### 

#### Crystal data

C_22_H_28_N_2_O_6_
*M* *_r_* = 416.46
Monoclinic, 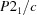

*a* = 8.267 (1) Å
*b* = 7.994 (1) Å
*c* = 17.582 (3) Å
β = 98.98 (2)°
*V* = 1147.7 (3) Å^3^
*Z* = 2
Mo *K*α radiation
μ = 0.09 mm^−1^
*T* = 173 K
0.50 × 0.04 × 0.01 mm

#### Data collection

Bruker–Nonius KappaCCD diffractometer
Absorption correction: multi-scan (*SADABS*; Bruker, 2001 ▶) *T*_min_ = 0.957, *T*_max_ = 0.999
11093 measured reflections
2626 independent reflections
1258 reflections with *I* > 2σ(*I*)
*R*_int_ = 0.096

#### Refinement

*R*[*F* ^2^ > 2σ(*F* ^2^)] = 0.055
*wR*(*F* ^2^) = 0.134
*S* = 0.93
2626 reflections
142 parameters
H atoms treated by a mixture of independent and constrained refinement
Δρ_max_ = 0.19 e Å^−3^
Δρ_min_ = −0.20 e Å^−3^

### 

Data collection: *COLLECT* (Nonius, 1999 ▶); cell refinement: *DIRAX/LSQ* (Duisenberg *et al.*, 2000 ▶); data reduction: *EVALCCD* (Duisenberg *et al.*, 2003 ▶); program(s) used to solve structure: *SIR97* (Altomare *et al.*, 1999 ▶); program(s) used to refine structure: *SHELXL97* (Sheldrick, 2008 ▶); molecular graphics: *ORTEP-3 for Windows* (Farrugia, 2012 ▶) and *Mercury* (Macrae *et al.*, 2006 ▶); software used to prepare material for publication: *WinGX* (Farrugia, 2012 ▶).

## Supplementary Material

Crystal structure: contains datablock(s) global, I. DOI: 10.1107/S160053681302480X/ds2234sup1.cif

Structure factors: contains datablock(s) I. DOI: 10.1107/S160053681302480X/ds2234Isup2.hkl

Additional supplementary materials:  crystallographic information; 3D view; checkCIF report

## Figures and Tables

**Table 1:** Hydrogen-bond geometry (Å, °)

*D*—H⋯*A*	*D*—H	H⋯*A*	*D*⋯*A*	*D*—H⋯*A*
N1—H1*A*⋯O2^i^	0.83 (2)	2.11 (2)	2.935 (3)	171 (2)
N1—H1*B*⋯O1^ii^	0.84 (2)	2.34 (2)	3.066 (2)	144 (2)
N1—H1*B*⋯O2	0.84 (2)	2.41 (2)	2.942 (3)	122.2 (18)

Symmetry codes: (i) 
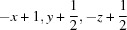
; (ii) 
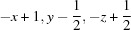
.

**Fig. 1.**
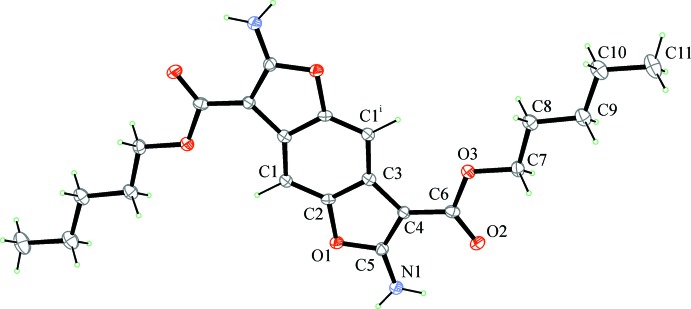
ORTEP view of the title compound. Thermal ellipsoids are drawn at 30% probability level.

**Fig. 2.**
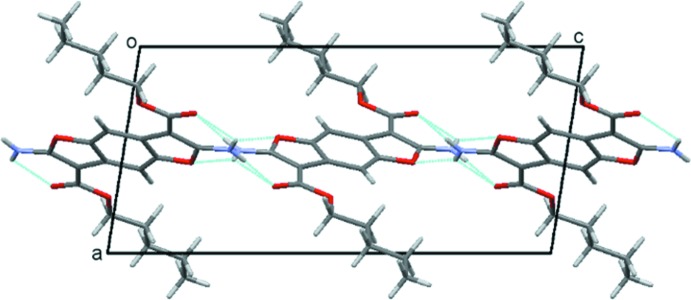
Crystal packing viewed along b axis. Hydrogen bonds are drawn as dashed lines.

## supplementary crystallographic information

### 1. Comment

In the field of our studies on the synthesis and properties of aminobenzodifurane derivatives (Caruso *et al.* 2009) we prepared the title compound, C_22_H_28_N_2_O_6_. The presence of an aromatic heterocyclic core in the molecule makes this kind of compound interesting in organic electronics (Carella *et al.* 2012; Centore *et al.* 2007). 2,6-diamino-benzo[1,2 - b;4,5 - b']difuran-3,7-dicarboxylic acid (Fig. 1) crystallizes in *P*2_1_/c space group with one half molecule in the independent unit. The molecule is located on a crystallographic inversion center and exhibits an all planar shape (maximum deviation from least square plane of the molecule is -0.0229 (17) for N1). The planarity of the molecule is a consequence of the all-*trans* conformation of penthyl groups and of the torsion angle at C6–O3 bond (C4—C6—O3—C7 = 179.7 (2)°). The planar conformation is also stabilized by intramolecular N—H···O=C hydrogen bonds (Table 1). In fact, the intramolecular hydrogen bonding involving the amino group and ester carbonyl helps to lock the *syn* conformation of ester with respect to amino group. The amino NH_2_ group is also involved in intermolecular hydrogen bonds, acting as donor towards benzodifurane oxigen (O1) and ester carbonyl oxygen (O2) acceptors (see Table 1). In the crystal packing (Fig. 2), molecules self-assemble into a two-dimensional hydrogen bonded network that display interdigital packing sustained by alkyl-alkyl interactions.

### 2. Experimental

2,6-diamino-benzo[1,2 - b;4,5 - b']difuran-3,7-dicarboxylic acid was prepared according to the procedure described in the literature (Caruso *et al.* 2009). Crystals suitable for X-ray analysis were obtained by slow evaporation of dioxane/water solution.

### 3. Refinement

All NH hydrogen atoms were located in difference Fourier maps and refined with *U*_iso_=1.2*U*_eq_(N) of the carrier atom. All the other H atoms were generated stereochemically and refined by the riding model with *U*_iso_=1.2×*U*_eq_ of the carrier atom (1.5 for H atoms of the methyl groups).

### Crystal data

**Table:**

C_22_H_28_N_2_O_6_	*F*(000) = 444
*M**_r_* = 416.46	*D*_x_ = 1.205 Mg m^−^^3^
Monoclinic, *P*2_1_/*c*	Mo *K*α radiation, λ = 0.71073 Å
Hall symbol: -P 2ybc	Cell parameters from 75 reflections
*a* = 8.267 (1) Å	θ = 3.1–16.9°
*b* = 7.994 (1) Å	µ = 0.09 mm^−^^1^
*c* = 17.582 (3) Å	*T* = 173 K
β = 98.98 (2)°	Block, grey
*V* = 1147.7 (3) Å^3^	0.50 × 0.04 × 0.01 mm
*Z* = 2	

### Data collection

**Table:**

Bruker–Nonius KappaCCD diffractometer	2626 independent reflections
Radiation source: normal-focus sealed tube	1258 reflections with *I* > 2σ(*I*)
Graphite monochromator	*R*_int_ = 0.096
Detector resolution: 9 pixels mm^-1^	θ_max_ = 27.5°, θ_min_ = 3.2°
CCD rotation images, thick slices scans	*h* = −10→10
Absorption correction: multi-scan (*SADABS*; Bruker, 2001)	*k* = −10→9
*T*_min_ = 0.957, *T*_max_ = 0.999	*l* = −22→22
11093 measured reflections	

### Refinement

**Table:**

Refinement on *F*^2^	Primary atom site location: structure-invariant direct methods
Least-squares matrix: full	Secondary atom site location: difference Fourier map
*R*[*F*^2^ > 2σ(*F*^2^)] = 0.055	Hydrogen site location: inferred from neighbouring sites
*wR*(*F*^2^) = 0.134	H atoms treated by a mixture of independent and constrained refinement
*S* = 0.93	*w* = 1/[σ^2^(*F*_o_^2^) + (0.061*P*)^2^] where *P* = (*F*_o_^2^ + 2*F*_c_^2^)/3
2626 reflections	(Δ/σ)_max_ < 0.001
142 parameters	Δρ_max_ = 0.19 e Å^−^^3^
0 restraints	Δρ_min_ = −0.20 e Å^−^^3^

### Special details

**Table:**

Geometry. All e.s.d.'s (except the e.s.d. in the dihedral angle between two l.s. planes) are estimated using the full covariance matrix. The cell e.s.d.'s are taken into account individually in the estimation of e.s.d.'s in distances, angles and torsion angles; correlations between e.s.d.'s in cell parameters are only used when they are defined by crystal symmetry. An approximate (isotropic) treatment of cell e.s.d.'s is used for estimating e.s.d.'s involving l.s. planes.
Refinement. Refinement of *F*^2^ against ALL reflections. The weighted *R*-factor *wR* and goodness of fit *S* are based on *F*^2^, conventional *R*-factors *R* are based on *F*, with *F* set to zero for negative *F*^2^. The threshold expression of *F*^2^ > σ(*F*^2^) is used only for calculating *R*-factors(gt) *etc*. and is not relevant to the choice of reflections for refinement. *R*-factors based on *F*^2^ are statistically about twice as large as those based on *F*, and *R*- factors based on ALL data will be even larger.

### Fractional atomic coordinates and isotropic or equivalent isotropic displacement parameters (Å^2^)

**Table:**

	*x*	*y*	*z*	*U*_iso_*/*U*_eq_	
C1	0.5923 (2)	0.1341 (3)	0.04464 (11)	0.0257 (5)	
H1	0.6556	0.2314	0.0731	0.026*	
C2	0.5354 (2)	−0.0094 (3)	0.07682 (11)	0.0246 (5)	
C3	0.4461 (2)	−0.1426 (3)	0.03695 (11)	0.0255 (5)	
C4	0.4171 (2)	−0.2648 (3)	0.09645 (11)	0.0254 (5)	
C5	0.4884 (3)	−0.1957 (3)	0.16650 (12)	0.0282 (5)	
C6	0.3356 (2)	−0.4269 (3)	0.09113 (12)	0.0288 (6)	
C7	0.1878 (3)	−0.6355 (3)	0.00858 (11)	0.0326 (6)	
H7A	0.0935	−0.6356	0.0370	0.033*	
H7B	0.2644	−0.7250	0.0301	0.033*	
C8	0.1290 (3)	−0.6679 (3)	−0.07634 (12)	0.0340 (6)	
H8A	0.2242	−0.6700	−0.1043	0.034*	
H8B	0.0556	−0.5760	−0.0979	0.034*	
C9	0.0363 (3)	−0.8366 (3)	−0.08811 (12)	0.0382 (6)	
H9A	0.1101	−0.9271	−0.0654	0.038*	
H9B	−0.0583	−0.8331	−0.0598	0.038*	
C10	−0.0261 (3)	−0.8793 (3)	−0.17302 (13)	0.0472 (7)	
H10A	0.0678	−0.8822	−0.2017	0.047*	
H10B	−0.1019	−0.7904	−0.1958	0.047*	
C11	−0.1155 (4)	−1.0494 (3)	−0.18205 (16)	0.0632 (8)	
H11A	−0.1539	−1.0711	−0.2367	0.063*	
H11B	−0.2094	−1.0467	−0.1542	0.063*	
H11C	−0.0400	−1.1383	−0.1609	0.063*	
N1	0.4973 (3)	−0.2479 (3)	0.23935 (11)	0.0383 (6)	
H1A	0.550 (3)	−0.192 (3)	0.2748 (12)	0.038*	
H1B	0.454 (3)	−0.340 (3)	0.2485 (12)	0.038*	
O1	0.56177 (16)	−0.04310 (18)	0.15705 (7)	0.0292 (4)	
O2	0.32315 (19)	−0.51874 (18)	0.14725 (8)	0.0377 (4)	
O3	0.27143 (17)	−0.47150 (17)	0.01739 (8)	0.0324 (4)	

### Atomic displacement parameters (Å^2^)

**Table:**

	*U*^11^	*U*^22^	*U*^33^	*U*^12^	*U*^13^	*U*^23^
C1	0.0243 (13)	0.0246 (14)	0.0275 (12)	0.0019 (10)	0.0017 (10)	−0.0028 (10)
C2	0.0267 (12)	0.0283 (14)	0.0184 (11)	0.0051 (11)	0.0018 (9)	0.0001 (10)
C3	0.0225 (12)	0.0268 (14)	0.0271 (12)	0.0040 (10)	0.0039 (10)	−0.0032 (10)
C4	0.0263 (12)	0.0216 (13)	0.0271 (12)	0.0021 (10)	0.0009 (10)	−0.0009 (10)
C5	0.0289 (13)	0.0252 (15)	0.0303 (13)	0.0008 (11)	0.0039 (10)	0.0012 (11)
C6	0.0260 (13)	0.0305 (15)	0.0286 (13)	0.0076 (11)	−0.0001 (10)	−0.0006 (11)
C7	0.0304 (13)	0.0269 (14)	0.0400 (14)	−0.0019 (11)	0.0041 (11)	−0.0014 (11)
C8	0.0282 (13)	0.0345 (16)	0.0385 (13)	0.0024 (11)	0.0029 (11)	−0.0043 (11)
C9	0.0292 (13)	0.0355 (16)	0.0487 (15)	0.0032 (11)	0.0025 (12)	−0.0065 (12)
C10	0.0432 (16)	0.0499 (18)	0.0483 (15)	−0.0056 (14)	0.0068 (13)	−0.0135 (13)
C11	0.0620 (19)	0.054 (2)	0.073 (2)	−0.0181 (16)	0.0085 (16)	−0.0242 (15)
N1	0.0530 (14)	0.0320 (14)	0.0268 (12)	−0.0109 (11)	−0.0033 (10)	0.0022 (10)
O1	0.0350 (9)	0.0296 (10)	0.0224 (8)	−0.0013 (7)	0.0025 (7)	0.0006 (7)
O2	0.0489 (10)	0.0313 (10)	0.0307 (9)	−0.0033 (8)	−0.0003 (7)	0.0075 (7)
O3	0.0373 (9)	0.0293 (10)	0.0289 (9)	−0.0043 (7)	0.0001 (7)	−0.0010 (7)

### Geometric parameters (Å, º)

**Table:**

C1—C2	1.393 (3)	C7—H7B	0.9900
C1—C3^i^	1.422 (3)	C8—C9	1.549 (3)
C1—H1	1.0231	C8—H8A	0.9900
C2—C3	1.418 (3)	C8—H8B	0.9900
C2—O1	1.419 (2)	C9—C10	1.540 (3)
C3—C1^i^	1.422 (3)	C9—H9A	0.9900
C3—C4	1.477 (3)	C9—H9B	0.9900
C4—C5	1.394 (3)	C10—C11	1.543 (3)
C4—C6	1.457 (3)	C10—H10A	0.9900
C5—N1	1.338 (3)	C10—H10B	0.9900
C5—O1	1.384 (2)	C11—H11A	0.9800
C6—O2	1.247 (2)	C11—H11B	0.9800
C6—O3	1.369 (2)	C11—H11C	0.9800
C7—O3	1.479 (2)	N1—H1A	0.83 (2)
C7—C8	1.518 (3)	N1—H1B	0.84 (2)
C7—H7A	0.9900		
			
C2—C1—C3^i^	114.40 (18)	C7—C8—H8B	109.5
C2—C1—H1	127.3	C9—C8—H8B	109.5
C3^i^—C1—H1	118.3	H8A—C8—H8B	108.1
C1—C2—C3	126.93 (18)	C10—C9—C8	114.01 (19)
C1—C2—O1	123.44 (18)	C10—C9—H9A	108.8
C3—C2—O1	109.63 (17)	C8—C9—H9A	108.8
C2—C3—C1^i^	118.67 (18)	C10—C9—H9B	108.8
C2—C3—C4	106.02 (17)	C8—C9—H9B	108.8
C1^i^—C3—C4	135.31 (19)	H9A—C9—H9B	107.6
C5—C4—C6	122.45 (19)	C9—C10—C11	112.2 (2)
C5—C4—C3	105.72 (18)	C9—C10—H10A	109.2
C6—C4—C3	131.82 (18)	C11—C10—H10A	109.2
N1—C5—O1	115.50 (19)	C9—C10—H10B	109.2
N1—C5—C4	132.4 (2)	C11—C10—H10B	109.2
O1—C5—C4	112.08 (18)	H10A—C10—H10B	107.9
O2—C6—O3	121.9 (2)	C10—C11—H11A	109.5
O2—C6—C4	124.58 (19)	C10—C11—H11B	109.5
O3—C6—C4	113.54 (18)	H11A—C11—H11B	109.5
O3—C7—C8	109.05 (16)	C10—C11—H11C	109.5
O3—C7—H7A	109.9	H11A—C11—H11C	109.5
C8—C7—H7A	109.9	H11B—C11—H11C	109.5
O3—C7—H7B	109.9	C5—N1—H1A	119.4 (15)
C8—C7—H7B	109.9	C5—N1—H1B	119.5 (15)
H7A—C7—H7B	108.3	H1A—N1—H1B	121 (2)
C7—C8—C9	110.81 (18)	C5—O1—C2	106.54 (16)
C7—C8—H8A	109.5	C6—O3—C7	115.84 (15)
C9—C8—H8A	109.5		
			
C3^i^—C1—C2—C3	0.1 (3)	C5—C4—C6—O2	−0.8 (3)
C3^i^—C1—C2—O1	179.41 (17)	C3—C4—C6—O2	178.5 (2)
C1—C2—C3—C1^i^	−0.1 (3)	C5—C4—C6—O3	179.06 (18)
O1—C2—C3—C1^i^	−179.49 (16)	C3—C4—C6—O3	−1.6 (3)
C1—C2—C3—C4	179.22 (19)	O3—C7—C8—C9	−178.43 (16)
O1—C2—C3—C4	−0.2 (2)	C7—C8—C9—C10	−179.49 (19)
C2—C3—C4—C5	0.7 (2)	C8—C9—C10—C11	179.2 (2)
C1^i^—C3—C4—C5	179.8 (2)	N1—C5—O1—C2	−178.64 (17)
C2—C3—C4—C6	−178.7 (2)	C4—C5—O1—C2	0.8 (2)
C1^i^—C3—C4—C6	0.4 (4)	C1—C2—O1—C5	−179.79 (18)
C6—C4—C5—N1	−2.1 (4)	C3—C2—O1—C5	−0.3 (2)
C3—C4—C5—N1	178.4 (2)	O2—C6—O3—C7	−0.4 (3)
C6—C4—C5—O1	178.55 (17)	C4—C6—O3—C7	179.72 (16)
C3—C4—C5—O1	−0.9 (2)	C8—C7—O3—C6	−178.24 (17)

Symmetry code: (i) −*x*+1, −*y*, −*z*.

### Hydrogen-bond geometry (Å, º)

**Table:**

*D*—H···*A*	*D*—H	H···*A*	*D*···*A*	*D*—H···*A*
N1—H1*A*···O2^ii^	0.83 (2)	2.11 (2)	2.935 (3)	171 (2)
N1—H1*B*···O1^iii^	0.84 (2)	2.34 (2)	3.066 (2)	144 (2)
N1—H1*B*···O2	0.84 (2)	2.41 (2)	2.942 (3)	122.2 (18)

Symmetry codes: (ii) −*x*+1, *y*+1/2, −*z*+1/2; (iii) −*x*+1, *y*−1/2, −*z*+1/2.
